# Impact of Packaging Methods on Physicochemical Properties, Flavor Profile, and Microbial Community in Low-Temperature Stored Mianning Ham

**DOI:** 10.3390/foods14132336

**Published:** 2025-07-01

**Authors:** Lin Chen, Mengdie Li, Yiting Song, Wei Wang, Jiamin Zhang, Ting Bai, Ling Gan, Congxia Tang, Lili Ji

**Affiliations:** 1 Sichuan Key Laboratory of Meat Processing, Chengdu University, Chengdu 610106, China; chenlin@cdu.edu.cn (L.C.); limengdie2000@163.com (M.L.); songyiting1996@hotmail.com (Y.S.);; 2 College of Veterinary Medicine, Southwest University, Chongqing 402460, China; 3 A’ba Tuyouda Agricultural Technology Co., Ltd., A’ba 624500, China

**Keywords:** Mianning ham, packing method, physical and chemical properties, volatile flavor compounds, microbial diversity

## Abstract

This study aims to determine the differences in the effects of vacuum packaging and modified atmosphere packaging on the quality, flavor, and microorganisms of Mianning ham. Vacuum packaging exhibits stronger antioxidant properties (a* value), while modified atmosphere packaging inhibits microorganisms and delays the decline of Aw through CO_2_. A total of 249 volatile substances was determined in the ham, while 19 main flavor substances, such as 1-octanol, hexanal, 2-nonanone, and p-cresol, were identified. It was found that the packaging method significantly affected the contents of alcohols and hydrocarbons. At the phylum level, Firmicutes is the dominant bacterial community. At the genus level, in the vacuum packaging group, *Tetragenococcus* and *Carnobacterium* are the core contributing bacteria for flavor, while *Staphylococcus* is dominant in both packaging types and may inhibit flavor formation.

## 1. Introduction

Dry-cured ham is a traditional fermented meat product with a long history of more than 1200 years [1]. The distinct flavors of dry-cured ham vary across different countries and regions due to factors, such as the diverse breeds of pigs, their feed, weight, age, and variations in production techniques [2]. Mianning ham originated in Mianning County, Liangshan Prefecture, Sichuan Province. This ham is carefully made from the hind legs of fat pigs after trimming, curing, cleaning, and fermentation processes [3]. Mianning ham is widely used as flavor enhancer, umami agent, and nutrition enhancer in Chinese food and dishes because of its bright color, pleasant flavor, rich nutrition, and good taste [4]. Mianning Ham has been protected by National Geographical Indications [5].

Mianning ham, as a traditional fermented meat product of Liangshan Prefecture, Sichuan Province, shares significant commonalities with other well-known fermented hams, such as Xuanwei ham, Jinhua ham, and Nuodeng ham, in terms of production techniques, microbial action, flavor formation, and nutritional value [6]. All traditional dry-cured hams rely on salt for dehydration, antibacterial effects, and preservation [7]. The flavor of dry-cured ham depends on the enzymatic hydrolysis of surface microorganisms. All dry-cured hams need to ferment and mature for several months to several years, as time promotes the deep hydrolysis of proteins and the accumulation of small-molecule flavor substances [8]. Dry-cured ham undergoes many enzymatic reactions during the long ripening process [9]. A study on Xuanwei ham found that the flavor substances of ham of different years are unique, which may be related to the metabolic activities of specific microorganisms in the ripening process [10]. However, fermented dry-cured meat products are generally not sterilized during the curing stage, and molds and microorganisms can thrive, leading to quality and safety problems for fermented dry-cured meat products [11]. Fermented dry-cured ham is sold at room temperature in Spain and other European countries [12]. Still, the quality of the fermented dry-cured ham will be reduced to a certain extent due to the microbial growth caused by the maturation of the ham when sold at room temperature [13]. Therefore, to extend the shelf life of ham products, researchers have studied different packaging methods, such as refrigeration, vacuum packaging, and modified atmosphere packaging (MAP) [14,15]. Among these technologies, MAP and vacuum packaging effectively prevent product contamination and moisture loss in meat products while extending their storage life [16].

In the modified atmosphere packaging of Iberian dry-cured ham, the color value of the 20% N_2_ + 80% CO_2_ group is more stable and the flavor is better [17]. Furthermore, +4 °C is a temperature above zero and is often used in the refrigerator’s fresh-keeping compartment. It can slow down the reproduction of microorganisms and keep food in a liquid state. On the other hand, −4 °C is a sub-zero low temperature. Water begins to freeze and some foods enter a semi-frozen state. It is suitable for light freezing [18]. This study chose slices of low-temperature Mianning ham as research subjects to investigate changes under two different packaging techniques, namely modified atmosphere packaging (20% N_2_ + 80% CO_2_) and vacuum packaging at a storage temperature range of ±4 °C. By evaluating physical and chemical parameters, volatile flavor compounds, and microbial diversity, we examined the quality variations in Mianning ham during low-temperature storage under different packaging methods. These findings provide valuable insights for future considerations regarding packaging and storage for Mianning ham.

## 2. Materials and Methods

### 2.1. Sample Preparation and Sampling

The samples used in this study were taken from Jiuyuan Ham Factory in Mianning County, Liangshan Prefecture. The hind leg of Liangshan Wujin pig was selected as the sample. Hybrid pigs are usually fed rice, buckwheat, and green feed. After feeding for 180 days, slaughter can be carried out when the live weight reaches 100 kg [19]. A total of 20 pigs’ hind legs that had been fermented for 1 year and 2 years, respectively, were selected. The samples of Mianning ham were vacuum-packed on site using a vacuum packaging machine (Witt-Gasetechnik GmbH Co., Witton, Germany) and then transported back to the laboratory.

After the hams were returned to the laboratory, the skin, fat, and bones of 20 hams were removed. The lean parts were selected and sliced (12 mm thick) through a Baijie 304 stainless steel slicer (Baijie, Beijing, China). The ham slices were randomly packaged using vacuum packaging and modified atmosphere packaging technology (Witt-Gasetechnik GmbH Co., Witton, Germany). Each experimental group comprised five replicate packages, with individual package weights standardized at 150 ± 2 g. The laminated film used for packaging consisted of a mixture of PA (polyamide) and PE (polyethylene) (Viduca, S.L., Valencian Community, Spain), with an oxygen transmission rate (OTR) of 38 cm^3^/m^2^/24 h/atm. Packages had a headspace volume ratio of 1:1. The ham slices were, respectively, vacuum-packed and modified atmosphere packaged. The vacuum packaging machine setting parameters were as follows: The pumping time was 40 s, the pressure was 0.2 MPa, and the heat sealing time was 5 s. The MAP machine setting parameters were as follows: The gas composition was 20% N_2_ + 80% CO_2_, the pressure was 0.04 MPa, the pumping time was 4 s, the charging time was 4 s, and the heat sealing time was 2 s. All samples were stored in darkness at ±4 °C. The physicochemical analysis of ham samples was conducted at storage intervals of 0, 25, 50, 75, and 100 days to investigate the dynamic changes in their physicochemical properties during low-temperature storage.

In the experiment, the following abbreviations were used: MA1 (modified atmosphere packaging at 4 °C for one year), MA2 (modified atmosphere packaging at 4 °C for two years), MB1 (modified atmosphere packaging at −4 °C for one year), MB2 (modified atmosphere packaging at −4 °C for two years), VA1 (vacuum packaging at 4 °C for one year), VA2 (vacuum packaging at 4 °C for two years), VB1 (vacuum packaging at −4 °C for one year), and VB2 (vacuum packaging at −4 °C for two years).

### 2.2. Measurement of Physical and Chemical Properties

After homogenizing the samples, the moisture content, water activity (Aw), pH, and malondialdehyde content were determined. Moisture content was determined using a CS-120H moisture tester (Guanya, Shenzhen, China), Aw was determined using a GYW-1 (Guanya, Shenzhen, China), and pH was determined by using a PHS-3C (Sanxin, Shanghai, China). Moisture content, malondialdehyde content, pH, and Aw were measured according to the method described by Wang et al. [20], where malondialdehyde was determined using spectrophotometry. Chromaticity values and the total viable count (TVC) were determined according to the method described by Li et al. [6] with a slight modification using a CR-400 portable colorimeter (Konica Minolta Co., Ltd., Tokyo, Japan), with a light source of D65, an observation angle of 10 °C, mirror surface component exclusion mode, and a hole diameter of 8 mm, using a standard color plate for whiteboard correction. Luminance (L*-value), redness (a*-value), and yellowness (b*-value) were recorded. All indicators were measured three times.

For the determination of the microstructure of ham, refer to Liu et al. [21] for the ZEISSGeminiSEM300 scanning electron microscopy (ZEISS Microscopy Co., Ltd., Shanghai, China) testing procedure. A surgical knife was used to take a cube sample with an edge length of 5 mm × 5 mm × 5 mm along the muscle fiber direction of the ham slice sample. It was then fixed in a 2.5% glutaraldehyde fixative solution for 24 h. The sample was rinsed three times with phosphate buffer solution, each rinse lasting 15 min. It was then dehydrated sequentially with ethanol solutions of 50%, 70%, and 90% volume fractions. For each dehydration step (15 min), the sample was transferred to a vacuum freeze dryer for a 12-h freeze-drying after the dehydration was completed. Then, gold was sprayed at a current of 15 mA for 90 s, with a gold spraying thickness of 10 nm. The microstructure was observed and photographed under a scanning electron microscope at a voltage of 15 kV.

### 2.3. Determination of Flavor Substances

The determination of volatile flavor substances was modified by referring to Chen et al. [5]. Solid phase microextraction (SPME) was used to extract volatile flavor substances. Pretreatment conditions were as follows: After 100 days of storage, samples from different treatment groups were minced, respectively, using the MJ-JC012 meat grinder (Midea, Guangdong, China). Then, 3.00 g samples were precisely weighed and placed in a headspace bottle with a capacity of 15 mL, and 1 μL of 2,4,6-trimethylpyridium was added as the referenced standard. The pretreatment conditions of the samples were set with the CTC automatic sampler, as follows: the temperature of the heater box was 75 °C, the heating time was 45 min, the sample extraction time was 20 min, and the analysis time was 5 min. Gas chromatography conditions (Agilent Technologies Co., Ltd., New York, NY, USA) were as follows: a HP-5MS UI gas chromatography column (30 mm × 0.25 mm, 0.25 mm) was used; the pressure was 32.0 kPa, the flow rate was 1.0 mL/min, the carrier gas was helium, and the sample is not divided. The inlet temperature was 250 °C. For the column temperature program, the initial temperature was 40 °C, for 20 min; the temperature was increased to 180 °C at 6 °C/min; then, the temperature was raised to 260 °C at a rate of 3 °C/min for 5 min. Conditions for mass spectrometry (Agilent Technologies Co., Ltd., New York, NY, USA) were as follows: an electron ionization source was used. The electron energy was 70 eV; the ion source temperature was 230 °C; the temperature of four-stage rod was 150 °C; the quality scanning range was 40~500 m/z; the detector voltage was 350 v.

The chromatogram was used to search and match in NIST for qualitative comparison, and volatile compounds with chromatogram peak matching degree of 80% were selected. The absolute contents of volatile flavor components were obtained according to the internal standard, the absolute contents of volatile flavor compounds were calculated according to the following Equation (1):

(1) $$C=\frac{C_{1}\times V\times A}{A_{1}\times m}$$

where *C* (μg/kg) is the absolute content of each volatile flavor compound; *C*_1_ (μg/μL) is the mass concentration of the internal standard; *V* (μL) is the added volume of the internal standard. *A* is the peak area of each volatile flavor compound; *A*_1_ is the peak area of internal standard; *m* (g) is the sample mass.

The key volatile flavor compounds were calculated by the odor activity value (*OAV*) method according to the following Equation (2):

(2) $$OAV=\frac{B_{1}}{B_{2}}$$

where *B*_1_ (μg/kg) is the content of compound in the sample; *B*_2_ (μg/kg) is the odor threshold of the compound.

### 2.4. High-Throughput Sequencing

All microbial DNA sequencing, PCR amplification, purification, and sequencing of Mianning ham samples were commissioned by Majorbio Co., Ltd. (Shanghai, China). Specific primers 341 (F) (5′-CCTACGGGNGGCWGCAG-3′) and 806 (R) (5′-GGACTACHVGGGTATCTAAT-3′) were used to amplify the V3–V4 region of bacteria by PCR [22]. The first PCR amplification system (50 μL) included the following: 5 µL of 10×KOD buffer, 5 µL of 2 mM dNTPs, 3 µL of 25 mM MgSO_4_, 1.5 µL of 10 µM forward primer, 1.5 µL of 10 µM reverse primers, 1 µL of KOD enzyme, and 100 ng of DNA template, with the rest filled with distilled water. The amplification procedure was set as follows: predenaturation at 94 °C for 2 min, denaturation at 98 °C for 10 s, annealing at 62~66 °C for 30 s, extension at 68 °C for 30 s and 30 cycles, and extension at 68 °C for 5 min. The second round amplification system (50 μL) was configured as follows: 5 µL of 10 × KOD buffer, 5 µL of 2 mM dNTPs, 3 µL of 25 mM MgSO_4_, 1 µL of 10 µM splice primer, 1 µL of 10 µM PCR universal primers, 1 µL of KOD enzyme, and 100 ng of DNA template, with distilled water to complete the total volume. The amplification procedure was set as follows: predenaturation at 94 °C for 2 min, denaturation at 98 °C for 10 s, annealing at 65 °C for 30 s, extension at 68 °C for 30 s and 12 cycles, and extension at 68 °C for 5 min. For methods, refer to Piotrowska-Cyplik and Shan et al. [23,24]. Referring to the preliminary quantitative results of electrophoresis, the PCR products were detected and quantified by the QuantiFluor™-ST blue fluorescence quantitative system.

Amplicons were collected from 2% agarose gel, purified according to the manufacturer’s instructions using an AxyPrep DNA gel extraction kit (Axygen Biotechnology Co., Ltd., Hangzhou, China), and quantified using an ABI StepOnePlus real-time PCR system. To ensure sequencing data quality, raw reads were processed using fastp (v0.18.0) [3,8] on 19 December 2023. This involved removing reads containing ≥10% unknown nucleotides (N), discarding reads with ≥50% bases having a Phred quality score ≤ 20, and removing reads containing adapters. The resulting clean reads were used for assembly. Paired-end clean reads were merged using FLASH (v1.2.11) with a minimum overlap of 10 bp and a maximum mismatch density of 2% [25]. Clean tags were obtained by filtering low-quality tags according to Puente-Sánchez et al.’s criteria in [26]. Clean tags were clustered into operational taxonomic units (OTUs) at ≥97% similarity using the UPARSE pipeline (v9.2.64) [27,28]. Potential chimeras were detected and removed using the UCHIME algorithm [29] (for 16S analysis), yielding valid tags [30]. These valid tags were clustered at 97% similarity, and the most abundant sequence within each OTU was selected as its representative sequence. Representative OTU sequences were taxonomically classified using the naive Bayes classifier within the RDP classifier (v2.2) against the SILVA database (v138) [31,32], with a confidence threshold of 0.8, to annotate bacterial 16S rRNA gene sequences.

### 2.5. Statistical Analysis

Excel 2021 (Microsoft, Redmond, WA, USA) was used for statistical data processing, SPSS Statistics 27.0 (IBM, Chicago, IL, USA) for analysis of variance (ANOVA). Origin 2022 was used for plotting broken line analysis charts and heat clustering plots, while R language was used for drawing heat clustering plots. All experiments were conducted independently three times, and the results were expressed as the mean ± standard deviation (SD).

## 3. Results and Analysis

### 3.1. Analysis of Physical and Chemical Results

**Figure 1:**
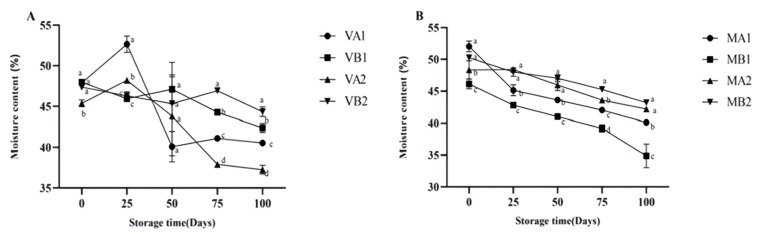
Changes in moisture content in different packaging methods of Mianning ham at the low-temperature storage stage: vacuum group (**A**) and modified atmosphere group (**B**). Note: MA1: modified atmosphere packaging at 4 °C for one year, MA2: modified atmosphere packaging at 4 °C for two years, MB1: modified atmosphere packaging at −4 °C for one year, MB2: modified atmosphere packaging at −4 °C for two years, VA1: vacuum packaging at 4 °C for one year, VA2: vacuum packaging at 4 °C for two years, VB1: vacuum packaging at −4 °C for one year, and VB2: vacuum packaging at −4 °C for two years. Lowercase letters indicate significant differences within the groups at the 0.05 level.

**Figure 2:**
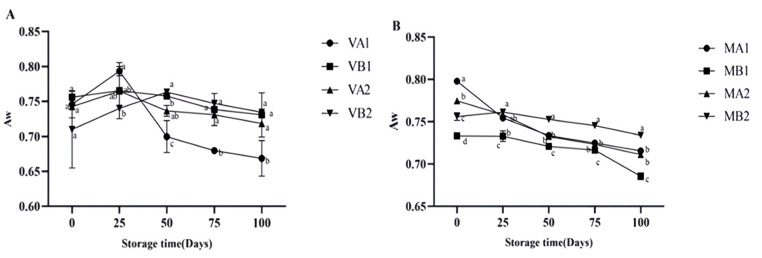
Changes in Aw in different packaging methods of Mianning ham at the low-temperature storage stage: vacuum group (**A**) and modified atmosphere group (**B**). Note: MA1: modified atmosphere packaging at 4 °C for one year, MA2: modified atmosphere packaging at 4 °C for two years, MB1: modified atmosphere packaging at −4 °C for one year, MB2: modified atmosphere packaging at −4 °C for two years, VA1: vacuum packaging at 4 °C for one year, VA2: vacuum packaging at 4 °C for two years, VB1: vacuum packaging at −4 °C for one year, and VB2: vacuum packaging at −4 °C for two years. Lowercase letters indicate significant differences within the groups at the 0.05 level.

The changes in the moisture content of Mianning ham under different packaging methods during low-temperature storage are depicted in Figure 1. In the vacuum packaging group, the moisture content of VA1 and VA2 increased initially and then decreased, while VB1 and VB2 both exhibited a downward trend. The overall trend in the modified atmosphere packaging group was downward. Unlike vacuum packaging, the fermented dry-cured ham in modified atmosphere packaging is exposed to gases during storage. The reduction in moisture content of dry-cured ham in modified atmosphere packaging is mainly caused by the evaporation and diffusion of moisture driven by the humidity difference between the dry gas environment inside the packaging and the surface of the ham [33]. Among the samples, MB1 reached the lowest level in 100 days, which was lower than MA1, MA2, and MB2 (*p* < 0.05). In modified atmosphere packaging, the degrees of moisture content change in MA1 compared with MB1 and that of MA2 compared with MB2 are quite different, indicating that the storage temperature will affect the moisture content change in ham slices. The changes in Aw are shown in Figure 2. Overall, the vacuum group showed an initial increase followed by a decrease. Specifically, the water activity of 1-year-old 4 °C ham (VA1) peaked on the 25th day and gradually decreased to 0.659 at 100 days. The modified atmosphere packaging group showed an overall downward trend. In modified atmosphere packaging, the Aw of group MB1 was the lowest. When MA1 was compared with MB1 and MA2 was compared with MB2, it was found that the degree of Aw change was gentle, indicating that the storage temperature in modified atmosphere packaging had a relatively small influence on the Aw of ham slices. Studies have shown that ham is susceptible to microbial contamination when the Aw is higher than 0.8, so reducing Aw is beneficial to preserve ham [34].

**Figure 3:**
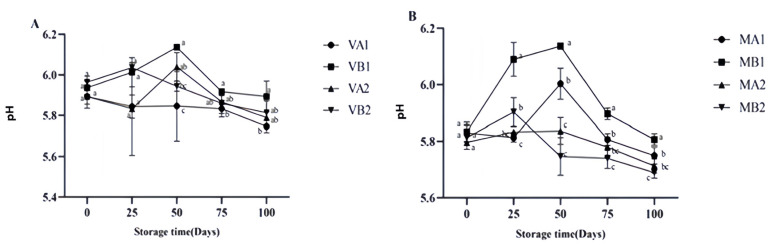
Changes in pH in different packaging methods of Mianning ham at the low-temperature storage stage: vacuum group (**A**) and modified atmosphere group (**B**). Note: MA1: modified atmosphere packaging at 4 °C for one year, MA2: modified atmosphere packaging at 4 °C for two years, MB1: modified atmosphere packaging at −4 °C for one year, MB2: modified atmosphere packaging at −4 °C for two years, VA1: vacuum packaging at 4 °C for one year, VA2: vacuum packaging at 4 °C for two years, VB1: vacuum packaging at −4 °C for one year, and VB2: vacuum packaging at −4 °C for two years. Lowercase letters indicate significant differences within the groups at the 0.05 level.

The pH value of dry-cured ham can serve as an indicator to determine its freshness and degree of spoilage [35]. The changes in the pH of Mianning ham under different packaging methods during low-temperature storage are presented in Figure 3. In the vacuum group, VB1 and VB2 showed an initial increase followed by a decrease, with VB1 reaching its peak at 50 days. In the modified atmosphere packaging group, the pH values of MB1 and MB2 increased initially and then decreased. MB1 peaked on the 50th day, the same trend as the vacuum group, and then continued to decline. At 100 days, the pH values of MB2, MA2, and VA1 were approximately 5.6, consistent with Parra et al.’s research results [16].

**Figure 4:**
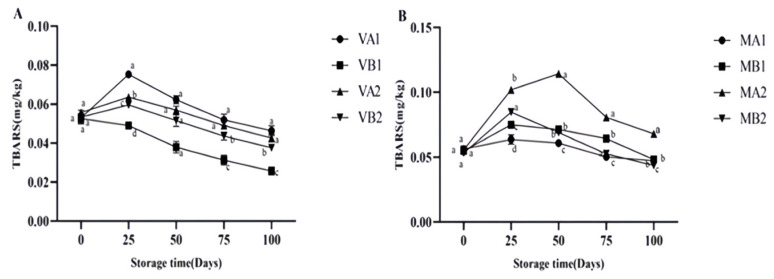
Changes in TBARS in different packaging methods of Mianning ham at the low-temperature storage stage: vacuum group (**A**) and modified atmosphere group (**B**). Note: MA1: modified atmosphere packaging at 4 °C for one year, MA2: modified atmosphere packaging at 4 °C for two years, MB1: modified atmosphere packaging at −4 °C for one year, MB2: modified atmosphere packaging at −4 °C for two years, VA1: vacuum packaging at 4 °C for one year, VA2: vacuum packaging at 4 °C for two years, VB1: vacuum packaging at −4 °C for one year, and VB2: vacuum packaging at −4 °C for two years. Lowercase letters indicate significant differences within the groups at the 0.05 level.

Malondialdehyde is the most abundant active aldehyde among the secondary lipid oxidation products during meat storage, which is a marker of lipid oxidative stress [4]. The thiobarbiturate reactant (TBARS) value obtained by measuring the malondialdehyde content of lipid oxidation products is a standard method to judge the degree of fat oxidation [8]. The greater the TBARS value of meat, the higher the degree of fat oxidation and the more significant the rancidity [36,37]. Figure 4 shows the changes in the TBARS of Mianning ham under different packaging methods during low-temperature storage. The vacuum group showed an initial increase followed by a decrease. In 25 days, all groups reached their peak overall, with VA1 having the highest value (0.075 mg/kg) at 25 days and VB1 having the lowest value (0.025 mg/kg) at 100 days. The content of VB1 was significantly lower than the other three groups (*p* < 0.05), indicating that the 1-year ham stored in a vacuum at −4 °C has the highest antioxidant capacity. The TBARS content in the modified atmosphere packaging group showed an initial increase followed by a decrease during storage. MA2 peaked (0.114 mg/kg) at 50 days and then showed a downward trend. MA2 (0.068 mg/kg) was the group with the highest content among the eight groups at 100 days, while the content changes for MA1, MB1, and MB2 were stable on the 100th day. According to Parra et al.’s study, the TBARS value of Iberian ham in different packaging methods showed an increasing trend during storage. Among them, the TBARS value of ham slices in modified atmosphere packaging was the lowest at 0–120 days, similar to the experimental result [16].

**Figure 5:**
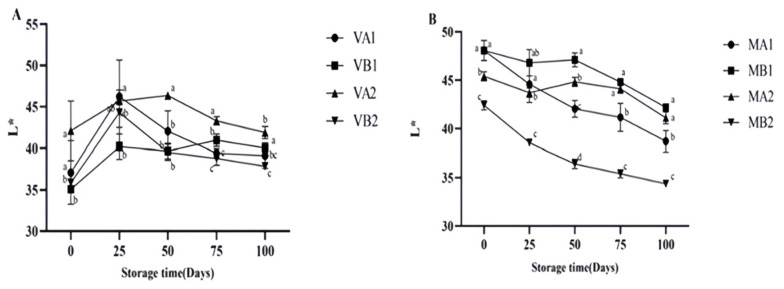
Changes in L* in different packaging methods of Mianning ham at the low-temperature storage stage: vacuum group (**A**) and modified atmosphere group (**B**). Note: MA1: modified atmosphere packaging at 4 °C for one year, MA2: modified atmosphere packaging at 4 °C for two years, MB1: modified atmosphere packaging at −4 °C for one year, MB2: modified atmosphere packaging at −4 °C for two years, VA1: vacuum packaging at 4 °C for one year, VA2: vacuum packaging at 4 °C for two years, VB1: vacuum packaging at −4 °C for one year, and VB2: vacuum packaging at −4 °C for two years. Lowercase letters indicate significant differences within the groups at the 0.05 level.

**Figure 6:**
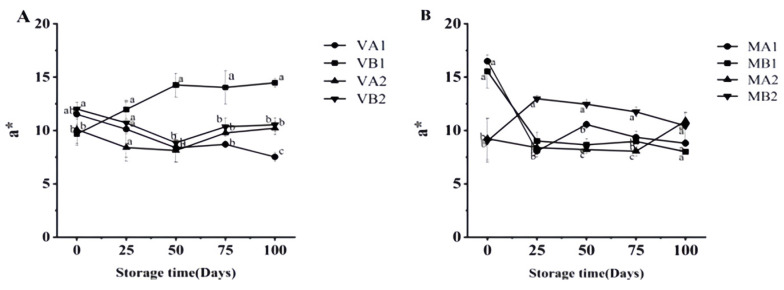
Changes in a* in different packaging methods of Mianning ham at the low-temperature storage stage: vacuum group (**A**) and modified atmosphere group (**B**). Note: MA1: modified atmosphere packaging at 4 °C for one year, MA2: modified atmosphere packaging at 4 °C for two years, MB1: modified atmosphere packaging at −4 °C for one year, MB2: modified atmosphere packaging at −4 °C for two years, VA1: vacuum packaging at 4 °C for one year, VA2: vacuum packaging at 4 °C for two years, VB1: vacuum packaging at −4 °C for one year, and VB2: vacuum packaging at −4 °C for two years. Lowercase letters indicate significant differences within the groups at the 0.05 level.

As can be seen from Figure 5, the lightness (L*) of Mianning ham shows that the L* of the vacuum group first increased and then decreased, among which the L* of ham at 4 °C in 2 years was the highest on the 50th day. The L* of the modified atmosphere group showed a downward trend, which may be caused by the enzymatic reaction during storage, consistent with the change in physical and chemical properties during the traditional processing of Jinhua ham [38]. At 100 d, the L* values of VB1, VB2, and VA1 were significantly different (*p* < 0.05). As seen from Figure 6 the vacuum group’s redness (a*) gradually increased, among which the a* of ham was highest at 100 days after a 1 year at −4 °C under vacuum. The larger the a* is, the redder the color of the ham is, and the a* of the ham stored under vacuum conditions is high. In modified atmosphere packaging, the a* of MA1 and MB1 was the largest at 0 days, and the a* decreased significantly after storage for 25 days. The reduction in a* might be due to the oxidation of myoglobin to form myoglobin, which turns the color of the meat brown [39]. Myoglobin (Mb) is the main source of the red color in meat products, and the oxidation state of the iron ions (Fe^2+^) in its center determines the color [40]. Oxymyoglobin (MbO_2_): Fe^2+^ binds to O_2_, presenting a bright red color (with a high a* value); Metmyoglobin (MetMb): Fe^3+^ without O_2_ binding, resulting in a brownish color (low a* value) [41]. Vacuum packaging: by completely removing oxygen, myoglobin is converted into deoxymyoglobin (purple–red), and the visual redness is reduced [42]. Modified atmosphere packaging: the O_2_ content is relatively low, and the lack of O_2_ hinders the formation of MbO_2_, while CO_2_ dissolves to form carbonic acid, lowering the pH and further promoting the oxidation of Fe^2+^ to Fe^3+^ [43]. These studies may be related to the reduction in a* in meat, and more in-depth research can be conducted subsequently.

**Figure 7:**
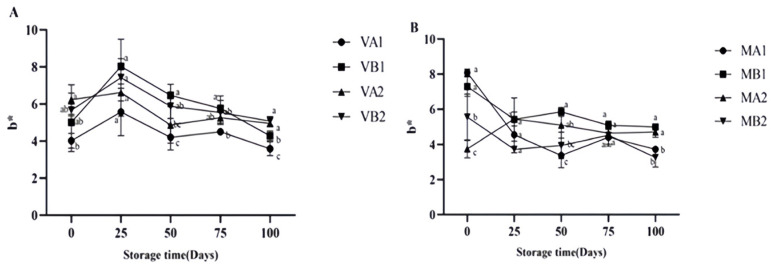
Changes in b* in different packaging methods of Mianning ham at the low-temperature storage stage: vacuum group (**A**) and modified atmosphere group (**B**). Note: MA1: modified atmosphere packaging at 4 °C for one year, MA2: modified atmosphere packaging at 4 °C for two years, MB1: modified atmosphere packaging at −4 °C for one year, MB2: modified atmosphere packaging at −4 °C for two years, VA1: vacuum packaging at 4 °C for one year, VA2: vacuum packaging at 4 °C for two years, VB1: vacuum packaging at −4 °C for one year, and VB2: vacuum packaging at −4 °C for two years. Lowercase letters indicate significant differences within the groups at the 0.05 level.

The yellowness (b*) in the vacuum group (Figure 7) first increased and then decreased, which may be due to the enzymatic reaction during storage, which is consistent with the research results of Zeng et al. [19]. Enzymatic reactions involve the combined action of endogenous enzymes (inherent in muscle tissue) and exogenous enzymes (secreted by microorganisms) [44]. Through pathways, such as protein degradation, lipid oxidation, and promoting the Maillard reaction, the composition of the coloring substances in ham is altered, ultimately manifesting as changes in the yellowness of its appearance [45]. The b* of the modified atmosphere group first decreased and gradually stabilized in the later storage stage. In the vacuum-packed group, it could be found that the one-year ham slices (VB1) stored at −4 degrees had better redness and yellowing, while the two-year ham slices (VA2) stored at 4 degrees had better brightness. In modified atmosphere packaging, both the MB1 and MB2 groups had better chromaticity, indicating that for modified atmosphere packaging, the influence of storage temperature on chromaticity is relatively small.

**Figure 8:**
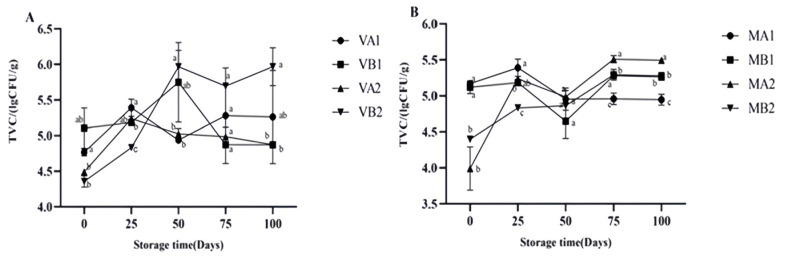
Changes in total viable count (TVC) in different packaging methods of Mianning ham at the low-temperature storage stage: vacuum group (**A**) and modified atmosphere group (**B**). Note: MA1: modified atmosphere packaging at 4 °C for one year, MA2: modified atmosphere packaging at 4 °C for two years, MB1: modified atmosphere packaging at −4 °C for one year, MB2: modified atmosphere packaging at −4 °C for two years, VA1: vacuum packaging at 4 °C for one year, VA2: vacuum packaging at 4 °C for two years, VB1: vacuum packaging at −4 °C for one year, and VB2: vacuum packaging at −4 °C for two years. Lowercase letters indicate significant differences within the groups at the 0.05 level.

The changes in the total viable count (TVC) of Mianning ham under different packaging methods during low-temperature storage are shown in Figure 8. The TVC in the vacuum group showed an initial increase followed by a decrease in 1-year-old and 2-year-old hams stored at −4 °C, with the 2-year-old ham stored at −4 °C having the highest TVC at 50 days. In vacuum packaging, VB2 consistently had a relatively high TVC after 50 days, while VA2 had a relatively low TVC after 50 days. As −4 °C is a frozen state, ice crystals piercing the cells may cause physical damage to the ham slices, leading to the leakage of cell contents and providing additional nutrients for the remaining cold-resistant microorganisms; on the other hand, 4 °C is the refrigeration state, and it mainly relies on low temperature to inhibit the growth of microorganisms. There is no large-scale cell damage, and only psychrophilic bacteria can grow, albeit extremely slowly [46]. Therefore, within a certain period of storage, the total number of colonies detected may instead be lower than that of the sample that has undergone the freezing process. MB2 in the modified atmosphere packaging group was the lowest at 25 days. This phenomenon may be because CO_2_ inhibits the growth of aerobic bacteria and extends the storage period of ham, which is consistent with the research viewpoint of Muhlisin et al. [47].

**Figure 9:**
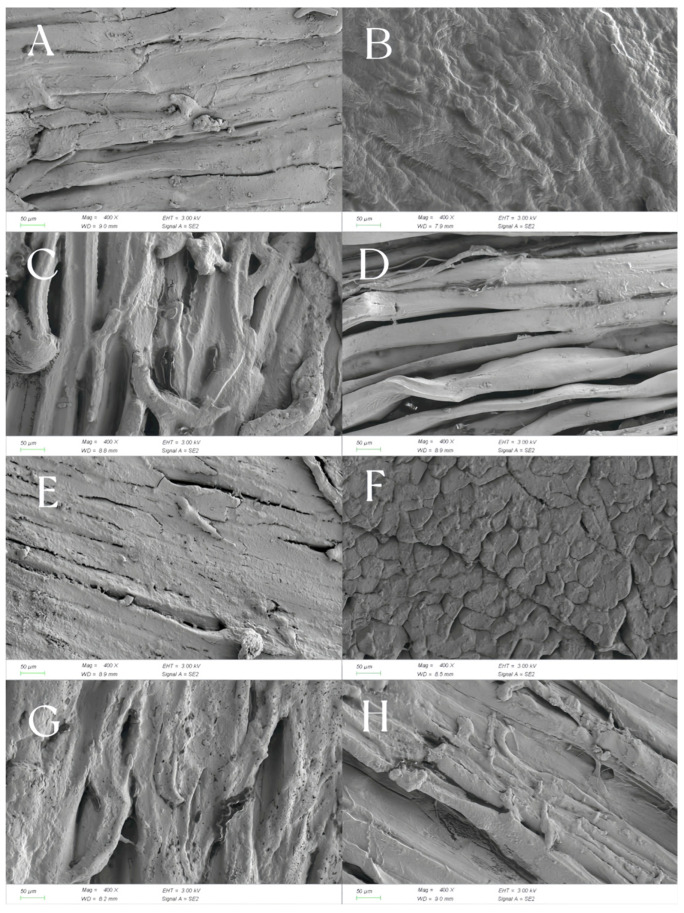
Microstructure of Mianning ham slices stored at low temperatures under different packing methods. Note: (1) (**A**) VA1 ham; (**B**) MA1 ham; (**C**) VB1 ham; (**D**) MB1 ham; (**E**) VA2 ham; (**F**) MA2 ham; (**G**) VB2 ham; (**H**) MB2 ham. (2) MA1: modified atmosphere packaging at 4 °C for one year, MA2: modified atmosphere packaging at 4 °C for two years, MB1: modified atmosphere packaging at −4 °C for one year, MB2: modified atmosphere packaging at −4 °C for two years, VA1: vacuum packaging at 4 °C for one year, VA2: vacuum packaging at 4 °C for two years, VB1: vacuum packaging at −4 °C for one year, and VB2: vacuum packaging at −4 °C for two years.

The microstructure changes in Mianning ham stored in different packaging methods at low temperatures are shown in Figure 9. As can be seen from Figure 9A,D,H, The muscle fibers are arranged in strips and have a relatively high degree of fiber integrity. The fibers in Figure 9A are wrinkled but continuous, while those in Figure 9D are parallel and compact. Figure 9C,G displays fiber dispersion and increased porosity. The interfiber voids in Figure 9C are obvious. The fibers in Figure 9G are looser and form pores. In Figure 9E, the fibers are broken or damaged. In Figure 9B, the surface is smooth and uniform, forming a dense structure. In Figure 9F, there are surface crack-like cracks. Therefore, the fibers in Figure 9A,D are intact and closely arranged, indicating that the ham has a firm texture. The samples in Figure 9C,G have loose fibers and many pores, indicating that the ham is relatively soft and loose. The surface cracking in Figure 9F indicates that the ham has poor water retention and a tough and hard texture. From the microstructure, it can be found that the ham in modified atmosphere packaging is tighter and stiffer, while the ham in vacuum packaging is softer and looser.

Structural differences may be linked to the curing process of hams: salt penetration dehydrates muscle tissue and induces protein denaturation, while suppressing spoilage microbes and preparing conditions for fermentation [48]. They may also stem from the fermentation stage: microorganisms, like lactic acid bacteria and molds, produce enzymes that break down proteins and fats, disrupting the fibrous structure and generating flavor compounds [49]. Moreover, moisture loss during storage impacts texture—excessive drying creates cracks, reducing the softness of the mouthfeel [50]. At present, there are relatively few studies on the changes in the microstructure of ham during storage. Subsequently, more detailed studies on the texture and taste of ham can be conducted in combination with the texture and storage environment.

#### Correlation Analysis of Physical and Chemical Properties

Table 1 presents the correlation analysis of physical and chemical indicators during the storage period of Mianning ham. It was found that the L* value and b* value were significantly positively correlated (0.655 **), indicating that the brightness of ham increased along with the yellowness. At the same time, L* was significantly negatively correlated with moisture content (−0.655 **), which confirmed that increasing moisture content would lead to color deepening. This finding is consistent with the research conclusions in the cured meat. Under high water content, muscle fibers absorb water and expand, the muscle structure becomes loose, the penetration depth of light increases while the reflection decreases, and, visually, the color becomes darker [51]. The a* value and pH value demonstrated a significantly positive correlation (0.679 **), i.e., an increase in pH leads to an enhancement of a*, possibly related to the stability of pigments, such as myoglobin or nitroso myoglobin. In fermented sausages, for example, a drop in pH can cause protein gelation, potentially damaging the pigment structure; a higher pH environment facilitates the binding of nitrite to myoglobin to form a stable red complex [52]. The decrease in pH value promotes protein gelation through isoelectric point aggregation, enhanced hydrophobicity and disulfide bond rearrangement [53]. At the same time, by protonation, the conjugated structure of pigment molecules is altered or their microenvironment is disrupted, resulting in color changes or fading [54]. The a* value and TBARS were significantly negatively correlated (−0.726 **), and the increase in malondialdehyde content was significantly correlated with the decrease in a*, indicating that the oxidation reaction led to the degradation of pigment. For example, free radicals produced by fat oxidation attack myoglobin, causing it to decolorize or convert to ferromyoglobin [55].

There was a significant positive correlation between moisture content and Aw (0.494 *), and the higher the moisture content, the higher the Aw. In sausages, samples with higher moisture content usually have higher Aw, which may be related to the influence of raw materials and processing technology in the moisture binding state [56]. Moisture content was significantly positively correlated with the TVC (0.412 *), and a high water environment promoted the growth of microorganisms, resulting in an increase in TVC. Reducing the moisture content is the key to inhibiting the TVC. For example, in the early stage of processing of fermented sausages, the moisture content is high, and lactic acid bacteria multiply rapidly, resulting in an increase in TVC. In the later stage, the moisture content decreases, inhibiting microbial activity, and the TVC decreases and tends to stabilize [57].

The pH was negatively correlated with malondialdehyde (−0.646 **), i.e., the malondialdehyde content increased when the pH decreased, possibly due to the acidic environment’s accelerated lipid oxidation or microbial activity [58]. Under acidic conditions, the lipid cell membrane is more easily hydrolyzed and polyunsaturated fatty acids are released as oxidation substrates [59]. In addition, metabolites produced by certain microorganisms (such as lactic acid bacteria) at low pH may have pro-oxidation effects [60].

### 3.2. Analysis of Volatile Flavor Substances

SPME-GC-MS detected a total of 249 kinds of volatile compounds during the low-temperature storage of Mianning ham in different packaging methods, including 49 alcohols, 36 esters, 15 aldehydes, 21 acids, 54 hydrocarbons, 39 ketones, and 35 other compounds (Supplemental Table S1). Among them, hydrocarbons were the most volatile flavor compounds in different packaging methods, followed by alcohols, ketones, and esters. As can be seen in the volatile flavor substance content statistics of Mianning ham in different packaging methods during the storage stage (Supplemental Table S2), the total absolute content of flavor substances in different packaging methods on the low-temperature storage stage was 11,831.4 μg/kg, while the total absolute content of alcohols and hydrocarbons in the eight groups of samples was higher (2776.25 μg/kg, 2400.91 μg/kg). The proportion of other compounds was the highest, reaching 1927.63 μg/kg. The highest aldehyde content was 419.52 μg/kg when the ham was stored at 4 °C for 100 days after being packaged in a modified atmosphere packaging for 1 year, and the lowest aldehyde content was 10.2 μg/kg in Mianning ham stored at −4 °C (VB1) after being packaged in a vacuum for 1 year. There were 46 kinds of acids in the vacuum group and 40 kinds in the modified atmosphere packaging group. The absolute content of hydrocarbons (561.16 μg/kg) was the highest in the 2-year storage stage of the modified atmosphere packaging at −4 °C. The threshold value of hydrocarbons is usually high, the odor is weak, and most of them are odorless, which has no noticeable effect on the flavor of ham [61]. The storage content of ketones in modified atmosphere packaging for 1 year at −4 °C reached 237.38 μg/kg. The highest value of esters was 242.72 μg/kg in modified atmosphere packaging at −4 °C for 1 year.

Odor activity value (OAV) can identify the main contributing volatile flavor substances. In order to further understand the differences in the contributions of volatile compounds to the overall flavor characteristics, the OAV of each compound is calculated based on the absolute content of each volatile flavor substance and the odor threshold (OTV) in the previous literature [3]. It is generally believed that when OAV ≥ 1, the contribution of volatile flavor compounds is proportional to OAV [19]. The larger the OAV, the more significant the contribution of this volatile flavor compound to the overall flavor [62]. The results are shown in Table 2. Based with the OAV value, 19 main flavor substances that affect the overall flavor characteristics of Mianning ham were screened out, including five alcohols, four ketones, five acids, one aldehyde, one ester, one phenol, and two pyrazines. Among them, 1-octanol, hexanal, 2-nonanone, and p-cresol contributed significantly to the flavor of Mianning ham in different packages. 1-Octanol has a strong oily smell and citrus aroma. Relevant studies have shown that 1-octanol has also been detected in Panxian ham [63]. The threshold of 1-octanol is relatively low, which may play an important role in the aroma characteristics of Mianning ham [3]. In long-aged hams, 1-octanol may participate in oxidation reactions to generate esters or acids, indirectly enhancing the fermented aroma and nutty aroma [64]. Hexanal is the main oxidation product in dry-cured meat, usually the result of linoleic acid degradation [65]. High concentrations of hexanal give off a rotten smell, while low concentrations of hexanal give off a grassy and vegetable flavor [66]. Hexanal has the highest OAV value in MA1, indicating that the ham will have a more intense grassy flavor under this packaging method. The OAV value in MB1 is relatively low, which might be due to the influence of different storage temperatures on hexanal [67]. Research on Iberian ham in Spain has found that 1-octanol and hexanal together form the distinctive “aged fat aroma” and “mushroom aroma” of the ham [68]. 2-Nonanone belongs to the ketone class of compounds, mainly derived from fat oxidation and microbial metabolism, especially significantly generated during the maturation stage of ham [69]. 2-Nonanone has a mild aroma similar to that nuts and cheese, which can enhance the richness and fermentation aroma of ham [62]. P-cresol belongs to phenolic compounds and is characterized by smoky and woody scents, mainly derived from the smoking process and microbial metabolism [70]. The processing of Mianning ham does not adopt the smoking technique [5]. Therefore, p-cresol should originate from microbial metabolism, that is, lactic acid bacteria and staphylococcus catalyze tyrosine or phenylalanine to generate phenolic derivatives through decarboxylase [71]. The nutty aroma of 2-nonanone and the smoky scent of p-cresol jointly build the complex flavor profile of the ham [68], Mianning ham has a nutty aroma under different processing conditions, indicating that 2-nonanone contributes significantly to the flavor of Mianning ham. Experiments show that both packaging methods can provide a richer aroma for the low-temperature-stored Mianning ham.

### 3.3. Analysis of Microbial Diversity

Alpha diversity refers to the species diversity within a single sample, covering two core dimensions, namely species richness (the number of species in the sample) and species evenness (the degree of balanced distribution of species in terms of quantity) [62]. The Chao index is an index for estimating the number of OTUs contained in a sample using the Chao 1 algorithm, and Chao 1 is often used in ecology to estimate the total number of species. The Ace index is an index used to estimate the number of OTUs in a community and is one of the commonly used indices for estimating the total number of species in ecology. The Simpson index is used to estimate one of the microbial diversity indices in a sample and is often used in ecology to quantitatively describe the biodiversity of a region; the larger the Simpson index value, the lower the community diversity is. The Shannon index is one of the microbial diversity indices used to estimate diversity in samples. It is similar to the Simpson diversity index and is often used to reflect the Alpha diversity index of the community; the larger the Shannon value, the higher the community diversity is. Good’s coverage refers to the coverage rate of each sample library; the higher the value, the higher the probability that the sequence in the sample is detected, and the lower the probability that it is not detected [3,19].

The Alpha diversity index is shown in Table 3. The microbial sequence coverage of Mianning ham of different groups was ≥0.99. This indicates that the amount of sequencing data was reasonable enough to analyze microbial information contained in different packaging methods of Mianning ham, and the accuracy of this analysis was high. As can be seen from the table, a total of 1061 OTUs were obtained by bacterial 16S division. The study showed that vacuum packaging retained more bacterial species in Mianning ham than modified atmosphere packaging, indicating a higher microbial diversity under vacuum conditions. The Shannon index and Simpson index can measure the diversity of microbial communities [3]. The Shannon index is usually between 1 and 10; the higher the score, the richer and more diverse the types of microorganisms are. The Simpson index is the opposite; the lower the score, the richer and more diverse the types of microorganisms are [72]. Specifically, group VA1 showed the most extensive Shannon index of 1.84 and the smallest Simpson index of 0.28, indicating that group VA1 had the most abundant bacterial diversity. The Simpson diversity index of the MA1 and MA2 groups was the highest, indicating that the community diversity was lower. The highest indexes of Chao and Ace appeared in the VB2 group, indicating that the microbial species and abundance were the highest in the VB2 storage stage. In contrast, the lowest indexes both appeared in MB2 and MA1. The results showed that the microbial species and abundance of Mianning ham in the modified atmosphere packaging were significantly lower than those in vacuum packaging, which may be because vacuum packaging provided an anaerobic environment, inhibited the growth of aerobic microorganisms, and limited oxidative rancidity. In addition, it was found that the types of microorganisms in modified atmosphere packaging were lower than those in vacuum packaging in dry-cured Iberian ham that had been refrigerated for a long time [16]. Subsequent research on dry-cured ham can be conducted in combination with the aspect of microbiology.

**Figure 10:**
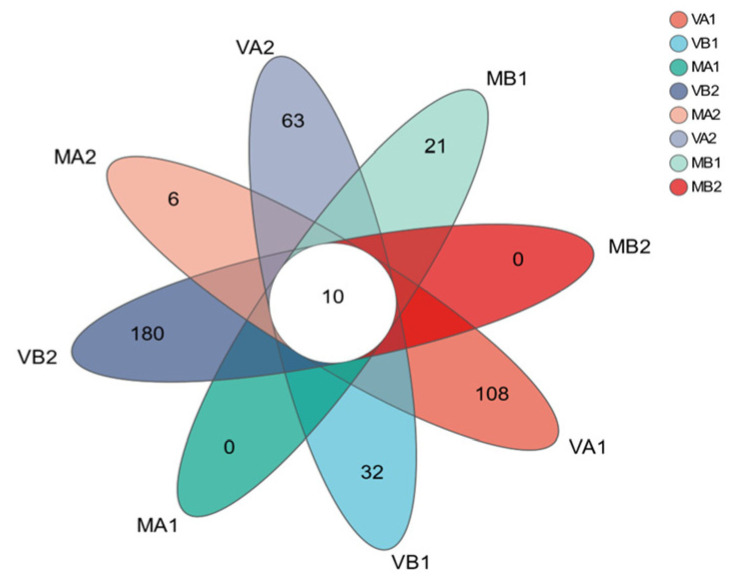
Venn diagram based on OTUs. Note: (1) Bacterial OUT distribution diagram; (2) MA1: modified atmosphere packaging at 4 °C for one year, MA2: modified atmosphere packaging at 4 °C for two years, MB1: modified atmosphere packaging at −4 °C for one year, MB2: modified atmosphere packaging at −4 °C for two years, VA1: vacuum packaging at 4 °C for one year, VA2: vacuum packaging at 4 °C for two years, VB1: vacuum packaging at −4 °C for one year, and VB2: vacuum packaging at −4 °C for two years.

The Venn plot based on OTUs is a very commonly used and intuitive visualization tool in microbiome research, especially in the analysis of bacterial communities [3]. It is mainly used to display the shared and unique distribution of bacterial OTUs among different samples or sample groups [62]. To understand the uniqueness, similarity, and overlap of microbial species composition of Mianning ham in different packaging methods at the low-temperature storage stage, the R language (version 3.3.1) tool was used for statistics and mapping, and Figure 10 was obtained. Other colors in the figure represent the different storage conditions of Mianning ham. It can be seen from the figure that there are 10 OTUs in the 8 groups of samples, indicating that the similarity level of bacterial diversity in the 8 groups is low. Among the 8 groups of samples, there are 180 bacterial OTUs in group VB2. At the same time, MA1 and MB2 have not detected the number of bacterial OTUs, and MA2 and MB1 have detected the most minor 6 and 21 OUT numbers, respectively. The results indicate that the types of specific microorganisms in the modified atmosphere group were much lower than those in the vacuum group. This might be because some of the CO_2_ in the modified atmosphere group was absorbed by the samples. CO_2_ can be absorbed and dissolved by meat tissues (mainly water and fat) [73]. The dissolved CO_2_ can easily enter the interior of microbial cells and undergo hydration to form carbonic acid and dissociation within the cells, leading to cytoplasmic acidification [35]. This indicates that CO_2_ has a certain influence on the microbial diversity of Mianning ham.

**Figure 11:**
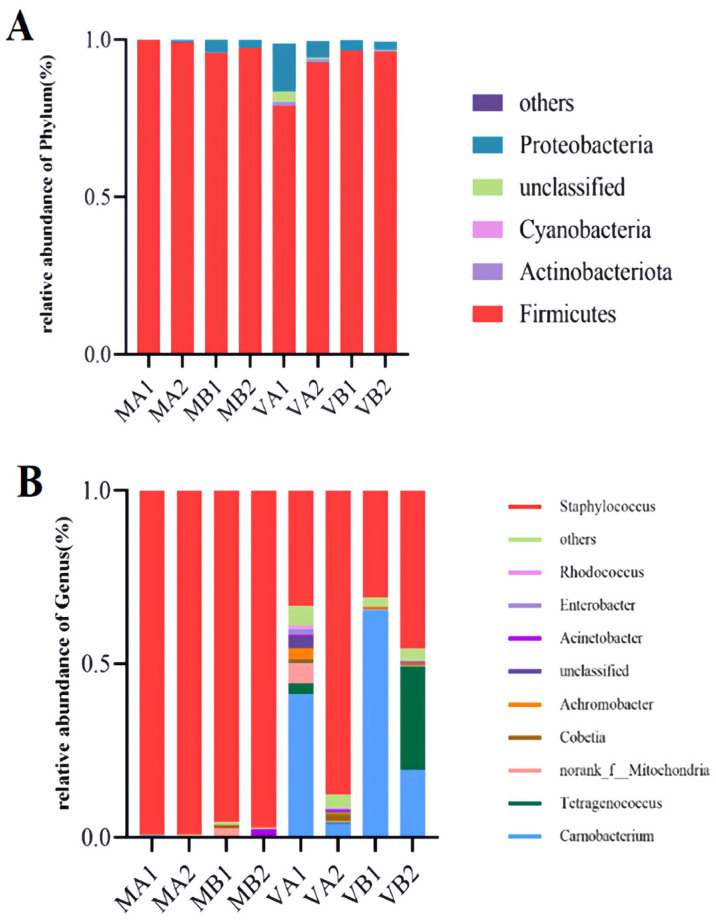
Species relative abundances of bacteria at phyla and genus levels (**A**,**B**). Note: (1) A—stacking diagram of bacteria at the phylum level, B—stacking diagram of bacteria at the genus level; (2) MA1: modified atmosphere packaging at 4 °C for one year, MA2: modified atmosphere packaging at 4 °C for two years, MB1: modified atmosphere packaging at −4 °C for one year, MB2: modified atmosphere packaging at −4 °C for two years, VA1: vacuum packaging at 4 °C for one year, VA2: vacuum packaging at 4 °C for two years, VB1: vacuum packaging at −4 °C for one year, and VB2: vacuum packaging at −4 °C for two years.

The stacking diagram can directly show microbial species composition in different Mianning ham packaging methods at the low-temperature storage stage. The top 10 microbial and bacterial genera with relative abundance were selected for in-depth study, and the other species with a relatively small number were collectively referred to as Others. According to Figure 11A, the four species with the highest abundance among the ten bacteria were Firmicutes, Proteobacteria, Cyanobacteria, and Actinobacteria, through the classification and analysis of the species. Firmicutes were the dominant bacterial species in the eight groups, of which MA1, MA2, MB2, and VB1 had the highest abundance (99.96%, 99.23%, 97.36%, and 96.25%, respectively). Proteobacteria had a high abundance of 15.19% in the VA1 group. Proteobacteria belong to Gram-negative bacteria as facultative anaerobic bacteria [62], and the relative abundance of Proteobacteria in the vacuum group is higher than that in the modified atmosphere group. The four cured meat products studied by Ongmu Bhutia et al. detected Firmicutes and Proteobacteria with the highest abundance, which was consistent with the results of this study [74].

It can be seen from Figure 11B that the top 10 microbial genera in relative abundance were identified at the genus level, which were *Staphylococcus*, *Rhodococcus*, *Enterobacter*, *Acinetobacter*, *Achromobacter*, *Cobetia*, *Tetragenococcus*, and *Carnobacterium*, respectively. Among them, *Staphylococcus* was the dominant bacterium in the modified atmosphere group, with the highest relative abundance. The relative abundance of this bacterium in MA1, MA2, MB1, and MB2 was 99.14%, 99.10%, 95.46% and 97.21%, respectively. *Staphylococcus* is a Gram-positive coccus, which may promote the growth of *Staphylococcus* in the atmosphere of 20% N_2_ + 80% CO_2_. During ham processing, *Staphylococcus* plays an active role in the formation of flavor by secreting protease and lipase, and the relative number of *Staphylococcus* is also the largest during the processing [75]. In the vacuum group, the levels of bacterial genera changed greatly, among which VA1 mainly included *Carnobacterium* and *Staphylococcus*, with relative abundances of 41.20% and 33.27%, respectively. The ham stored in a vacuum package at 4 °C for 1 year grew competitively in the absence of oxygen between *Carnobacterium* and *Staphylococcus*. In VA2, *Staphylococcus* was dominant, with a relative abundance of 87.64%. Compared with VA1, the two-year-old ham of the VA2 group had a lower moisture content. It is possible that *Staphylococcus* was more adapted to this environment and grew in large numbers, replacing *Carnobacterium*. In VB1, *Carnobacterium* became the dominant strain with a relative abundance of 65.43%. Compared with VA1 and VB2, it may grow in an environment of −4 °C with high moisture. In VB2, the dominant bacteria genera were *Staphylococcus*, *Tetragenococcus*, and *Carnobacterium*, and their relative abundances were 45.66%, 29.74%, and 19.51%, respectively. Among them, *Staphylococcus* and *Tetragenococcus* grew in large numbers. They competed with *Carnobacterium*, indicating that vacuum packaging of Mianning ham fermented for two years would greatly reduce the growth of *Carnobacterium*. In conclusion, *Staphylococcus*, *Tetragenococcus*, and *Carnobacterium* were the dominant bacteria in the vacuum group of Mianning ham under different packaging methods at the low-temperature storage stage. At the same time, *Staphylococcus* was dominant in the modified atmosphere group.

## 4. Correlation Analysis Between Microorganisms and Key Flavor Substances

**Figure 12:**
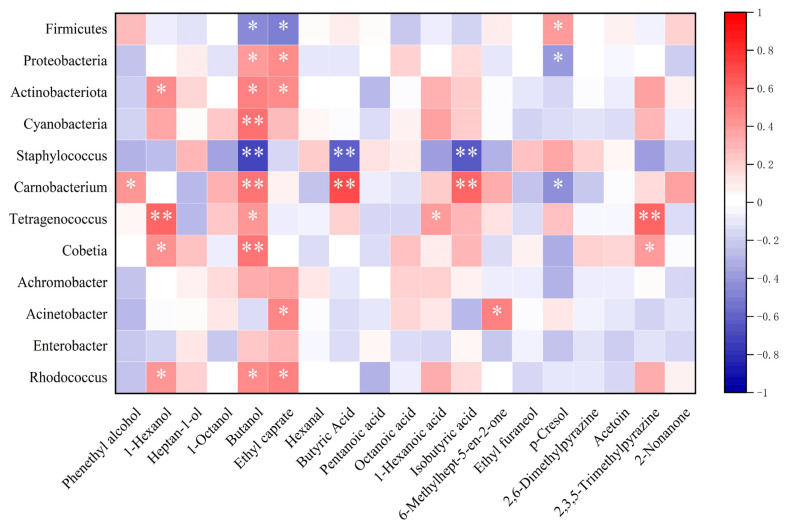
Heat map of correlation analysis between dominant microorganisms and key flavor compounds. Note: On the correlation heat map, the first four rows represent the correlation between the dominant phylum and key volatile compounds, while the last eight rows represent the correlation between the dominant genus and key volatile compounds. * significant correlation; ** extremely significant correlation.

Based on the analysis of dominant microorganisms and essential volatile flavor compounds in Mianning ham under varying packaging methods and storage temperatures, a thermal aggregation map was constructed to illustrate the correlations between microbial communities and flavor substances. As shown in Figure 12, at the phylum level, Firmicutes exhibited negative correlations with numerous key flavor compounds, whereas Proteobacteria, Cyanobacteria, and Actinobacteria demonstrated positive correlations with critical alcohol compounds influencing the ham’s flavor profile. This suggests that Firmicutes may inhibit flavor compound formation in Mianning ham.

Notably, *Tetragenococcus* showed a highly significant positive correlation with 1-hexanol and 2,3,5-trimethylpyrazine, while *Carnobacterium* was strongly positively correlated with butanol, butyric acid, and isobutyric acid. Among these, butanol displayed pronounced microbial dependency, being significantly associated with *Cyanobacteria*, *Carnobacterium*, and *Cobetia*. These findings indicate that *Tetragenococcus* and *Carnobacterium* likely play pivotal roles in flavor compound accumulation during ham storage. Conversely, *Staphylococcus* exhibited significant negative correlations with butanol, butyric acid, and isobutyric acid. Lower *Staphylococcus* content corresponded to higher concentrations of these flavor compounds, implying that *Staphylococcus* may suppress flavor development in Mianning ham. Herefore, *Carnobacterium* and *Tetragenococcus* appear essential for generating key flavor compounds in Mianning ham across different packaging and storage conditions.

## 5. Conclusions

The results show that the effects of different packaging methods on Mianning ham in terms of physical and chemical aspects are significantly different. Vacuum-packed ham has stronger antioxidant capacity, i.e., better retention of the a* value, while modified atmosphere packaged ham effectively inhibits the decrease in Aw and can inhibit the growth of microorganisms through CO_2_. A total of 249 volatile compounds in different packages of low-temperature stored Mianning ham were detected by SPME-GC-MS technology, and 19 key flavor substances were screened out. Among them, 1-octanol, hexanal, 2-nonanone, and p-cresol played important roles in the flavor formation of Mianning ham. Both modified atmosphere and vacuum packaging can enhance the flavor richness of the ham stored at low temperatures, but different packaging has a significant impact on the content of specific flavor substances. Using Illumina high-throughput sequencing technology, it was found that, at the phylum level, Firmicutes dominated, and at the genus level, the dominant bacteria in the vacuum packaging group were *Staphylococcus*, *Tetragenococcus*, and *Carnobacterium*. *Staphylococcus* is also the dominant bacterium in the modified atmosphere packaging group. *Staphylococcus* may inhibit the flavor of Mianning ham, while *Tetragenococcus* and *Carnobacterium* are indispensable contributions to the key flavor compounds of Mianning ham. Vacuum packaging can stabilize the quality of the ham, while modified atmosphere packaging provides a safe environment for preservation. Therefore, enterprises need to flexibly choose based on product positioning, storage conditions, and consumption scenarios, and simultaneously strengthen flavor control and standardized production. In future research, a combination of natural preservatives and packaging methods can be adopted: for instance, natural preservatives can be added to modified atmosphere packaging to enhance antibacterial effects in synergy with CO_2_ and reduce the use of chemical preservatives. The research results provide a theoretical basis for optimizing the packaging process and quality control of Mianning ham and have guiding significance for the development of the Mianning ham industry.

## Figures and Tables

**Table 1:** Correlation analysis of physicochemical indexes in the storage stage of ham. Note: * Significant correlation (*p* < 0.05); ** extremely significant correlation (*p* < 0.01).

Index	L*	a*	b*	Moisture Content	Aw	pH	TVC	TBARS
L*	1							
a*	0.129	1						
b*	0.655 **	0.065	1					
Moisture content	−0.655 **	0.169	−0.265	1				
Aw	−0.321	−0.033	0.067	0.494 *	1			
pH	0.34	0.679 **	0.395	−0.114	−0.036	1		
TVC	−0.287	−0.149	0.206	0.412 *	−0.031	−0.08	1	
TBARS	0.216	−0.726 **	0.071	−0.136	−0.307	−0.646 **	0.216	1

**Table 2:** Volatile compounds content of Mianning ham with different packaging methods and storage temperatures (μg/kg). Note: MA1: modified atmosphere packaging at 4 °C for one year, MA2: modified atmosphere packaging at 4 °C for two years, MB1: modified atmosphere packaging at −4 °C for one year, MB2: modified atmosphere packaging at −4 °C for two years, VA1: vacuum packaging at 4 °C for one year, VA2: vacuum packaging at 4 °C for two years, VB1: vacuum packaging at −4 °C for one year, and VB2: vacuum packaging at −4 °C for two years.

Compounds	OTV (μg/kg)	OAV Value (OAV ≥ 1)							
		VA1	VA2	VB1	VB2	MA1	MA2	MB1	MB2
Phenethyl alcohol	0.045	-	156.12	458.32	215.61	194.00	47.67	249.52	-
1-Hexanol	0.2	112.97	219.03	96.65	230.56	57.35	29.33	84.99	77.82
Heptan-1-ol	0.2	58.31	83.99	-	-	81.97	39.18	-	40.90
1-Octanol	0.054	730.15	795.28	1252.72	999.66	729.45	394.49	194.49	1302.59
Butanol	0.5	306.16	299.56	266.57	291.30	-	12.34	-	-
Hexanal	0.21	643.75	531.36	-	299.79	1760.64	354.27	8.85	600.10
Ethyl caprate	0.02	501.64	35.76	-	12.14	20.60	20.14	-	372.28
6-Methylhept-5-en-2-one	1	5.07	8.89	17.91	9.57	3.68	4.39	-	17.33
Acetoin	10	1.48	3.04	4.28	3.11	1.17	-	10.74	2.02
2-Nonanone	0.05	83.03	761.11	1142.95	272.85	287.97	495.30	8.06	202.20
Butyric Acid	2.73	17.20	37.79	22.81	23.68	1.44	2.26	37.70	16.74
Pentanoic acid	0.5	4.51	3.60	4.70	2.49	7.25	4.86	6.09	3.67
Octanoic acid	5	1.72	1.29	1.34	-	1.45	0.95	3.73	2.12
1-Hexanoic acid	1.5	13.97	17.30	16.35	15.86	11.85	5.21	-	16.41
Isobutyric acid	1	5.89	6.57	10.96	7.34	4.66	-	4.93	-
Ethyl furaneol	0.0025	511.64	-	-	-	146.58	84.74	8674.84	477.59
P-cresol	0.002	-	642.93	703.74	262.57	107.69	365.97	-	141.14
2,6-Dimethylpyrazine	10	2.04	3.20	2.02	2.80	-	-	22.35	1.69
2,3,5-Trimethylpyrazine	0.071	170.39	320.21	214.62	388.30	-	38.05	227.01	-

**Table 3:** Bacterial 16S rDNA Alpha diversity index. Note: MA1: modified atmosphere packaging at 4 °C for one year, MA2: modified atmosphere packaging at 4 °C for two years, MB1: modified atmosphere packaging at −4 °C for one year, MB2: modified atmosphere packaging at −4 °C for two years, VA1: vacuum packaging at 4 °C for one year, VA2: vacuum packaging at 4 °C for two years, VB1: vacuum packaging at −4 °C for one year, and VB2: vacuum packaging at −4 °C for two years.

Group	OUTs	Shannon Index	Simpson	Chao Index	Ace Index	Good’s Coverage
VA1	165.66 ± 8.50	1.84 ± 0.11	0.28 ± 0.02	174.97 ± 3.90	175.15 ± 8.56	0.99 ± 0.00
VB1	102.66 ± 50.50	0.67 ± 0.19	0.69 ± 0.12	118.48 ± 53.97	118.87 ± 55.53	1.00 ± 0.00
VA2	179.66 ± 28.71	1.29 ± 0.22	0.45 ± 0.06	215.98 ± 18.54	211.87 ± 20.28	0.99 ± 0.00
VB2	245.66 ± 16.25	1.55 ± 0.20	0.32 ± 0.07	282.65 ± 18.45	283.65 ± 17.67	0.99 ± 0.00
MA1	17 ± 3.60	0.05 ± 0.06	0.98 ± 0.02	25.62 ± 2.40	43.10 ± 16.15	0.99 ± 0.00
MB1	62.66 ± 18.03	0.23 ± 0.21	0.92 ± 0.10	82.03 ± 32.41	89.49 ± 38.87	0.99 ± 0.00
MA2	46.66 ± 6.80	0.07 ± 0.02	0.98 ± 0.00	67.39 ± 11.84	93.31 ± 31.60	0.99 ± 0.00
MB2	245.66 ± 16.25	0.12 ± 0.19	0.95 ± 0.09	16.11 ± 9.17	16.60 ± 8.91	1.00 ± 0.00

## Data Availability

The original contributions presented in the study are included in the article/Supplementary Material, further inquiries can be directed to the corresponding author.

## Supplementary Materials

The following supporting information can be downloaded at: https://www.mdpi.com/article/10.3390/foods14132336/s1, Table S1. Statistical analysis of the content of volatile flavor substances of Mianning ham under different packaging methods during low-temperature storage; Table S2. Volatile flavor substance content statistics of Mianning ham in different packaging methods during storage stage.
