# Supplementary material for: Impact of Packaging Methods on Physicochemical Properties, Flavor Profile, and Microbial Community in Low-Temperature Stored Mianning Ham
Source: Foods. 2025 Jul 1;14(13):2336. doi: 10.3390/foods14132336 (PMC12249448; doi:10.3390/foods14132336)
Supplement: Supplementary file 1 [file foods-14-02336-s001.zip › foods-3679888-supplementary.pdf]

Supplemental Table S1. Statistical analysis of the content of volatile flavor substances of Mianning ham under different packaging methods during low-temperature storage.

Number	Compound Name	Absolute Content (μg/kg)							
		MA1	MA2	MB1	MB2	VA1	VA2	VB1	VB2
	**Alcohols**								
1	Phenethyl alcohol	8.73±1.15	2.14±0.05	11.23±7.80	-	-	7.03±4.12	20.62±11.92	9.70±1.01
2	1- Hexyl alcohol	11.47±2.94	3.94±0.41	17.00±5.10	15.56±3.41	22.59±17.85	43.8±8.13	12.76±9.05	46.11±13.30
3	1-Heptanol	16.39±8.86	5.86±2.39	-	5.44±2.74	7.84±5.94	16.7±4.66	-	-
4	1- Capryl alcohol	39.39±13.30	21.3±12.95	7.02±2.03	70.34±17.11	39.43±28.33	42.9±5.95	24.99±9.76	9.82±2.80
5	Trans-2-Octen-1-ol	5.20±1.02	-	-	-	5.13±3.46	-	-	3.50±1.06
6	1-hexanol	11.47±2.40	5.87±1.74	17.01±5.60	15.56±4.84	22.59±8.43	43.81±10.1	19.33±1.91	46.11±2.61
7	(Z)-2-Octen-1-ol	9.56±0.29	5.29±2.62	1.58±0.73	14.60±1.97	4.34±2.79	9.03±1.20	8.98±5.87	6.85±3.09
8	1-Octen-3-ol	53.68±15.03	22.03±9.34	8.45±4.40	56.64±8.06	49.65±33.27	54.4±4.26	33.78±21.90	59.62±6.01
9	(S)-(+)-Propylene glycerol	3.55±2.00	-	-	-	-	-	13.67±6.87	-
10	2,3-Butanediol	96.58±17.42	24.42±2.23	-	172.66±49.77	43.92±18.36	24.8±11.5	-	13.91±7.23
11	1-Pentanol	16.80±8.77	8.24±2.50	16.38±0.23	-	-	23.92±3.44	-	4.53±2.03
12	5-Methyl-1-heptanol	5.35±1.01	-	13.68±4.92	6.82±4.98	-	-	-	-
13	Linalool	5.50±0.34	-	-	3.50±2.12	-	-	-	-
14	Methyl benzyl alcohol	2.25±0.51	2.51±1.19	-	3.11±2.76	4.02±3.49	4.75±1.14	-	-
15	-2,3-Butanediol	4.41±0.01	36.01±11.06	-	111.49±56.61	126.82±56.45	-	-	-
16	Cyclobutanol	-	1.93±0.61	-	-	-	-	-	1.77±0.59
17	Butanol	-	6.17±3.59	-	-	153.08±89.75	149.78±33.6	133.29±47.66	145.65±57.06
18	Butyleneglycol	-	-	1.42±0.53	39.07±0.53	21.42±19.65	7.1±1.6	11.28±2.65	9.45±4.18
19	Ethyl Alcohol	-	-	0.19±0.07	-	-	-	-	0.31±0.28
20	4-Hexanol, 1-methyl-	-	-	0.95±0.43	-	-	7.81±1.9	-	7.95±6.97
21	n-Hexanol	-	-	-	8.75±4.09	-	-	-	-
22	1-Azacyclic ethanol	-	-	-	3.87±2.36	-	-	-	-

23	3-Methyl-1-butanol	-	-	-	6.18±5.58	0.63±0.46	-	18.67±6.34	0.30±0.12
24	R(-)-2-pentanol	-	-	-	3.02±2.85	-	-	-	-
25	1-Hepten-4-ol	-	-	-	1.15±0.13	0.72±0.65	-	-	-
26	Hydrobenzoin	-	-	-	4.59±2.19	3.82±1.85	2.71±0.35	-	3.04±2.67
27	3-methylpentan-1-ol	-	-	-	5.26±2.38	-	-	-	-
28	1-phenylpropane-1,2-diol	-	-	-	-	1.52±0.45	-	-	-
29	Cycloheptanol	-	-	-	-	1.26±1.02	-	-	1.82±0.84
30	1-Propanol	-	-	-	-	1.77±0.80	-	5.04±2.37	2.62±1.89
31	DL-3-Methyl-2-butanol	-	-	-	-	1.23±1.01	-	-	-
32	Diisobutyl glycol	-	-	-	-	-	4.65±2.19	-	3.57±2.58
33	2-Methyl-5-Hexen-3-ol	-	-	-	-	-	2.64±0.83	-	-
34	1,12-Dodecanediol	-	-	-	-	-	3.29±1.03	-	-
35	(S)-(+)-5-Methyl-1-heptanol	-	-	-	-	-	4.80±1.34	-	-
36	3-Buten-2-ol	-	-	-	-	-	3.04±2.86	1.38±1.01	2.52±1.70
37	3,4-Dimethyl-1-Pentanol	-	-	-	-	-	4.3±1.7	-	-
38	Pentanol	-	-	-	-	-	-	4.32±3.88	3.09±2.90
39	Allyl alcohol	-	-	-	-	-	-	2.11±1.32	-
40	CIS-2-Penten-1-ol	-	-	-	-	-		3.18±2.91	-
41	4-Heptanol	-	-	-	-	-	-	1.57±0.52	3.20±1.97
42	(+/-)-4-Penten-2-ol	-	-	-	-	-	-	2.22±1.05	-
43	Isoacrylic acid	-	-	-	-	-	-	3.10±2.71	-
44	1-Pentanol	-	-	-	-	-	-	-	0.56±0.49
45	2-Ethyl-1,3-hexanediol, mixture of isomers	-	-	-	-	-	-	-	0.31±0.20
46	3-Ethyl-5-Hexen-3-ol	-	-	-	-	-	-	-	2.21±2.00
47	Alpha-phenylethyl alcohol	-	-	-	-	-	-	-	1.23±1.08
48	2-Methyl-3-hexanol	-	-	-	-	-	-	-	3.35±2.92
49	2-Methyl-1-propanol	-	-	-	-	-	-	-	0.33±0.09
	**Esters**								
1	Methyl isobutyrate	1.73±0.78	1.78±0.28	5.56±3.42	6.37±3.55	5.89±1.66	6.99±1.92	6.07±2.78	3.16±0.16

2	Di-N-Propyl Oxalate	2.02±1.15	3.15±0.70	-	-	-	-	-	-
3	Butyl propyl oxalate	1.77±1.02	-	-	-	7.41±2.74	0.29±0.06	-	14.31±10.84
4	ethyl caprate	0.41±0.28	0.41±0.25	-	7.45±2.50	10.03±6.95	0.71±0.03	-	0.24±0.14
5	Vinyl Hexanoate	7.92±2.49	3.49±1.47	3.12±1.78	3.52±0.67	3.93±1.70	4.48±1.95	-	4.82±1.29
6	gamma-hexalactone	3.83±1.00	-	24.26±6.08	4.09±2.93	6.59±3.74	3.25±2.82	2.96±1.63	3.08±2.69
7	Propylacrylate	2.48±1.00	-	-	0.39±0.14	-	38.0±24.2	0.50±0.14	1.61±0.42
8	Butyrolactone	3.88±0.47	2.47±1.25	0.28±0.04	10.74±3.21	9.35±4.11	10.0±8.69	10.93±3.64	13.38±4.18
9	Dienoic acid ester	-	-	98.61±17.13	-	-	-	-	-
10	Ethyl nitrite	10.22±0.67	21.67±10.4<br>4	-	-	7.34±5.37	-	20.72±15.01	-
11	Col acetate	1.34±0.22	1.10±0.08	-	-	-	-	-	-
12	Pentyl Formate	1.05±0.20	4.20±3.69	-	-	11.22±9.77	6.60±5.80	-	-
13	Allyl isobutyl oxalate	-	-	29.47±13.76	-	-	-	-	-
14	Isobutyl propyl oxalate	-	-	46.86±34.73	-	-	-	-	-
15	Methyl acetate	-	-	34.48±29.89	-	4.75±2.05	-	-	2.07±1.87
16	Diethyl carbonate	-	-	-	1.33±0.51	-	-	-	-
17	Esters	-	-	-	6.95±3.04	-	-	-	-
18	(-)-Menthyl Chloroformate	-	-	-	0.08±0.02	-	-	-	-
19	Hexenyl butyrate	-	-	-	3.14±1.98	-	-	-	-
20	Butyl Acrylate	0.66±0.03	-	-	-	0.66±0.48	-	-	-
21	2-propenyl formate	-	-	-	-	70.09±60.86	-	-	-
22	hexyl butyrate	-	-	-	-	1.44±1.15	3.29±1.11	-	2.37±1.08
23	Allyl heptyl oxalate	-	-	-	-	-	0.90±0.23	-	-
24	Butyl 6-ethyl-3-octyl phthalate	-	-	-	-	-	2.84±1.66	-	2.17±1.91
25	2-Ethoxyethyl 2-methoxycarbonate	-	-	-	-	-	4.14±3.71	-	3.63±1.16
26	Amyl 2-methylpropionate	-	-	-	-	-	9.42±2.86	-	11.66±10.25
27	1, 2-phthalic acid, di (2-methylpropyl) ester	-	-	-	-	-	1.73±0.62	-	-

28	Cyclohexyl isobutyl oxalate	-	-	-	-	-	-	3.35±1.03	-
29	Cyclobutyl isobutyl phthalate	-	-	-	-	-	-	2.02±0.92	2.64±1.34
30	1,2,4-benzenetricarboxylic acid, 1,2-diMethyl ester	-	-	-	-	-	-	3.92±2.53	-
31	Propyl valerate	-	-	-	-	-	-	4.17±3.63	-
32	Dibutyl phthalate	-	-	-	-	-	-	7.41±6.46	-
33	Ethyl butyrate	-	-	-	-	-	-	-	17.03±5.49
34	Butyl 2-methylbutyrate	-	-	-	-	-	-	-	23.67±21.11
35	propyl lactate	-	-	-	-	-	-	-	1.72±1.04
36	isocyanic acid	-	-	0.08±0.02	-	-	-	-	-
	**Ketone**					-			
1	2-Heptanone	5.92±0.02	-	-	-	5.26±4.05	-	54.61±27.32	-
2	Methylheptenone	3.68±0.39	4.39±2.55	-	4.02±1.84	5.07±3.62	8.88±1.55	12.16±4.58	9.57±4.06
3	4-Hydroxy-5-methyl-3-furanone	0.37±0.13	0.13±0.11	14.58±10.64	0.85±0.67	0.65±0.18	-	-	-
4	2,2,3-Trimethylcyclobutan-1-one	11.58±1.00	-	6.94±2.60	-	-	-	-	10.56±9.18
5	1-Octen-3-one	5.01±1.00	-	-	-	-	-	-	-
6	8-Nonen-2-one	4.11±1.28	2.28±1.28	-	-	7.95±4.01	8.85±7.80	8.88±7.70	16.07±4.49
7	Acetyl methyl carbinol	11.70±5.99	7.99±3.19	71.60±32.04	20.16±5.41	14.76±10.52	28.55±16.8	19.36±5.79	31.14±11.20
8	2,3-Octanedione	29.11±12.89	3.89±2.38	23.30±12.22	-	-	-	-	-
9	Acetone	21.25±4.75	10.76±9.80	0.11±0.06	9.29±4.94	10.70±2.21	33.3±28.9	-	19.68±17.07
10	Methyl vinyl ketone	1.91±0.09	2.09±1.92	-	7.49±6.51	-	-	-	-
11	2-Nonanone	14.40±1.76	24.76±7.99	0.40±0.14	10.11±5.96	4.15±1.14	38.0±14.2	57.15±33.29	13.64±4.85
12	Furanone	-	-	11.34±6.46	-	-	-	-	-
13	Strawberry furanone	-	-	97.59±39.09	-	-	-	-	-
14	2-Butanone, 3-methoxy-methyl- (6CI,7CI,9CI)	-	-	0.86±0.01	1.97±1.15	-	-	0.66±0.36	-
15	methylimidazolidine-2,4-dione	-	-	3.31±1.61	-	15.09±8.82	-	-	-
16	5-(bromomethyl)oxetan-2-	-	-	3.83±1.34	3.61±1.89	2.18±1.19	-	-	-

	one								
17	5-Hexen-2-one	-	-	-	5.25±3.93	8.82±6.78	-	-	-
18	Cyclobutanone	-	-	-	1.21±1.00	-	-	-	-
19	5-(Cyclohexylmethyl)pyrrolidin-2-one	-	-	-	1.38±0.38	-	-	-	-
20	2-acetoxyketoxime	-	-	-	9.27±6.05	-	-	-	-
21	fluoroketone	-	-	-	17.66±5.32	8.35±7.44	-	-	-
22	Butyroin	-	-	-	1.23±0.40	-	-	-	-
23	Hexanone	-	-	-	5.13±1.24	-	-	-	-
24	Cyclopropyl methyl ketone	-	-	-	5.10±3.64	-	-	-	0.52±0.25
25	Acetone	-	-	-	2.46±0.79	-	3.31±1.45	-	-
26	2,2,3-Trimethylcyclobutanone	-	-	-	-	18.79±8.08	-	2.98±0.89	8.84±5.45
27	4-methyl-4-heptene-3-one	-	-	-	-	3.23±2.87	-	-	-
28	Methoxyacetone	-	-	-	-	2.62±1.07	-	-	1.97±1.05
29	Methyl isopropenyl ketone	-	-	-	-	1.41±1.18	-	-	17.63±15.31
30	2, 4-imidazoldione, 5-methyl -	-	-	-	-	-	16.3±3.95	4.88±2.84	11.25±9.82
31	3,3-Dimethyl-2-Oxetanone	-	-	-	-	-	-	3.86±1.43	-
32	2,3-hexanedione	-	-	-	-	-	-	1.88±0.65	-
33	2,3-Butanedione	-	-	-	-	-	-	7.75±3.75	-
34	2,4(3H,5H)-Pyrimidinedione (9CI)，5-amino-6-nitro-	-	-	-	-	-	-	-	12.40±6.80
35	4-Hexen-2-one	-	-	-	-	-	-	-	20.81±18.07
36	Acetylacetone	-	-	3.52±1.13	-	4.80±2.49	-	-	-
37	trans-3-Penten-2-one	-	-	-	-	-	-	-	0.75±0.70
38	3-Methyldihydrofuran-2.5-dione	-	-	-	-	-	-	-	4.92±1.31
39	5-hydroxy-2, 7-dimethyl-4-octanone	-	-	-	-	-	-	-	12.44±3.24

	**Aldehyde**								
1	(Z) -2-decenal	1.43±1.00	-	-	-	-	-	-	-
2	(E)-2-Octenal	3.59±1.06	5.00±1.45	9.18±3.96	6.09±3.30	8.41±2.57	9.51±3.70	2.69±0.81	6.17±5.35
3	Hexanal	369.73±87.40	74.40±22.41	1.86±1.06	126.02±79.55	135.19±88.98	111.23±49.7	-	62.96±38.02
4	nona-2,4-dien-1-al	9.59±0.81	1.81±0.61	-	-	-	-	-	-
5	2-Methylvaleraldehyde	21.30±0.37	13.37±2.42	-	1.39±0.76	-	6.0±4.6	-	28.34±24.61
6	3-(methylthio)propionaldehyde	2.00±0.93	25.93±8.33	-	16.88±2.95	-	58.7±19.0	-	33.41±12.88
7	Decenal	1.90±0.93	15.93±5.18	-	-	-	-	-	-
8	3-Methylbutyraldehyde	4.21±0.86	13.87±1.82	19.45±7.51	95.89±51.73	-	118.77±13.12	-	-
9	alpha-ethyl phenylacetaldehyde	-	-	-	13.39±2.99	-	-	7.51±6.52	3.58±1.14
10	Heterocyclic aromatic aldehyde	-	-	-	10.03±1.79	-	-	-	-
11	Succinaldehyde	-	-	-	-	0.38±0.06	-	-	1.34±1.04
12	Acrolein	-	-	-	-	-	5.04±2.39	-	-
13	Ethyl methylphenylglycidate	-	-	-	-	-	-	-	1.94±0.93
14	2-methylhexal	-	-	-	-	-	-	-	7.21±2.48
15	2,4-decadienal	5.77±0.03	-	-	-	-	-	-	-
	**Acids**								
1	Butyric acid	3.94±1.48	6.18±1.94	102.92±22.12	45.70±15.28	46.96±16.19	103.17±23.12	62.26±13.25	64.65±14.81
2	N-Valeric Acid	23.62±2.66	52.66±1.73	13.04±0.54	11.24±1.24	1.34±1.09	1.21±0.36	1.56±0.35	1.25±0.91
3	Octanoic acid	7.24±1.77	4.77±1.31	18.64±7.3	10.6±3.65	8.6±3.26	6.45±3.81	6.7±3.88	-
4	Hexanoic acid	37.77±7.82	27.82±4.65	10.89±0.80	24.62±4.24	20.95±11.71	25.9±5.76	1.39±0.23	13.78±7.19
5	Valeric anhydride	4.59±2.00	-	8.18±2.11	-	-	14.87±2.00	-	-
6	Decanoic acid	22.50±0.88	31.88±0.74	13.28±1.41	19.22±3.78	5.04±1.73	3.43±1.07	0.65±0.12	1.40±1.04
7	D-Proline	35.80±5.00	-	-	-	-	-	-	-

8	Isovaleric acid	4.46±1.41	34.41±3.88	9.50±3.25	-	-	-	3.42±1.27	10.47±3.11
9	Isobutyric acid	4.66±3.00	-	4.93±2.93	-	5.89±3.43	6.56±1.18	10.96±6.68	7.34±3.26
10	Propyl pyruvate	21.90±0.99	13.99±0.77	-	13.68±3.22	-	-	-	-
11	2-methylhexanoic acid	14.70±0.25	20.25±8.88	-	10.35±3.45	19.23±12.91	48.9±14.7	2.22±1.83	9.25±2.61
12	Acetic acid	13.02±0.82	22.82±2.48	10.38±0.40	47.68±11.31	-	3.40±2.98	0.77±0.21	-
13	L-Alanine	-	-	3.75±2.41	-	-	-	-	-
14	N-Valeric Acid	-	-	-	14.93±4.70	-	1.79±1.05	1.29±0.15	2.50±1.33
15	2-Ethylheptanoic acid	-	-	-	-	11.18±1.27	-	-	0.62±0.09
16	4-Hydroxybutyric acid	-	-	-	-	1.91±0.85	38.76±8.75	-	-
17	Nonanoic acid	-	-	-	-	-	-	1.36±0.33	-
18	acetoxyacetic acid	-	-	-	-	-	-	1.98±0.54	2.50±0.51
19	4-Phenylbutyric acid	-	-	-	-	-	-	0.74±0.15	-
20	isopropyruvate	-	-	-	-	-	-	-	4.73±1.39
21	Alpha-ethyl cyclohexane acetic acid	-	-	-	-	-	-	-	0.79±0.59
	**Hydrocarbon**								
1	2,3-Dimethyl-1-hexene	12.89±2.27	4.27±3.91	-	-	-	-	-	-
2	1-pentanol	6.44±2.33	14.33±1.25	28.18±16.92	6.44±2.59	2.47±1.39	-	-	-
3	6-methyl-1-octene	3.09±0.23	2.23±1.02	-	3.46±1.25	-	-	-	-
4	3-propoxy-1-propene	-	-	11.91±6.50	-	-	-	-	-
5	3,4- Dimethyl-1-hexene	-	-	-	-	15.60±13.65	-	-	-
6	2-allyl cyclobutene	-	-	-	-	1.12±0.94	-	-	-
7	4,4-Dimethyl-1-pentene	-	-	-	-	2.16±1.10	3.19±2.77	-	-
8	3-Methyl-1-Hexene	-	-	-	-	-	6.33±5.66	-	-
9	8-Methyl-1-decene	-	-	-	-	-	1.93±1.14	-	-
10	Decane	103.66±24.18	47.18±23.73	8.14±3.74	206.67±23.24	187.68±64.58	39.3±25.0	194.61±70.19	69.35±30.09
11	n-Octane	2.36±1.28	3.28±2.47	7.22±3.50	4.56±1.10	6.12±4.24	12.0±3.03	5.59±2.85	108.53±13.32
12	3,6-Dimethyloctane	39.09±1.00	-	-	22.55±13.77	-	-	-	-

13	S-(-)-1,2-Epoxypropane	3.78±1.00	-	-	-	-	9.21±8.00	-	-
14	3, 7-dimethylnonane	4.16±0.02	-	-	-	-	-	3.19±2.90	-
15	Tetradecane	2.47±0.94	6.94±1.57	-	-	-	-	-	0.13±0.02
16	difluoromethane	-	-	-	7.97±3.74	-	-	-	-
17	Trimethylene oxide	12.30±0.12	1.92±1.22	-	-	-	-	-	1.76±0.61
18	1-nonane iodide	3.32±0.86	22.86±9.81	-	-	-	-	2.97±1.70	-
19	Trimethylethylene oxide	5.07±0.33	2.33±0.29	-	-	-	2.68±1.96	-	-
20	1-Chloropentane	3.01±0.69	2.69±1.39	-	-	-	-	-	-
21	1-Nitropropane	-	-	148.00±63.14	-	-	-	-	-
22	Dimethyl-undecane	-	-	24.60±12.36	50.51±9.59	-	-	-	-
23	n-Butyl isocyanide	-	-	23.41±4.04	-	-	-	-	-
24	Heterocyclic butane	-	-	47.48±12.95	-	-	-	-	-
25	Propoxybutane	-	-	3.23±0.81	-	-	-	-	-
26	1-iodododecane	-	-	4.61±2.02	4.08±3.54	-	3.86±2.39	-	-
27	6-ethyl-2-methyl-decane	-	-	3.09±1.59	-	-	-	-	-
28	Cyclopropane	-	-	0.19±0.10	-	-	-	-	-
29	Isobutane	-	-	17.79±10.43	-	-	-	-	-
30	3-Methylundecane	-	-	-	0.94±0.61	-	-	-	-
31	1, 3-epoxy-4-methylpentane	-	-	-	7.11±3.25	-	-	-	-
32	n-Undecane	-	-	-	0.52±0.25	-	-	-	-
33	Tetradecane iodide	-	-	-	46.50±20.28	-	-	-	13.72±11.92
34	Iododecane	-	-	-	53.46±36.30	10.03±0.01	3.48±1.11	2.94±1.62	-
35	3-bromohexane	-	-	-	1.33±0.30	-	-	-	-
36	Naphthenic	-	-	-	8.50±0.06	6.62±5.77	13.2±6.5	3.62±1.44	2.81±1.91
37	Alkane	-	-	-	61.08±22.90	-	-	-	-
38	Haloalkanes	-	-	-	0.58±0.23	0.98±0.66	-	-	-
39	Methyltridecane	-	-	-	0.99±0.35	0.49±0.24	-	-	1.53±0.67
40	2-Methylbutane	-	-	-	27.17±5.79	-	-	-	-
41	1-isobutylcyclopropyl	-	-	-	42.84±29.68	13.14±3.52	-	-	-

42	3-Methylnonane	-	-	-	-	14.48±2.60	35.0±10.4	-	-
43	2-nitropentane	-	-	-	-	35.61±20.96	-	-	-
44	3,3-Dimethylhexane	-	-	-	-	45.03±9.03	81.7±10.8	-	4.95±2.64
45	2,2-Dimethylbutane	-	-	-	-	53.38±18.10	-	-	-
46	2-Bromopentane	-	-	-	-	-	12.54±2.51	-	-
47	Tetramethylethylene oxide	-	-	-	-	-	11.46±1.51	-	-
48	n-Octane，2,4,6-Trimethyl-	-	-	-	-	-	13.01±1.13	-	-
49	2-Ethoxypropane	-	-	-	-	-	-	0.78±0.29	0.88±0.23
50	Trimethylcyclopentane	-	-	-	-	-	-	-	13.52±3.83
51	2,3,3-Trimethylpentane	-	-	-	-	-	-	-	53.56±37.38
52	Methane isocyanate	-	-	-	-	-	33.1±18.7	-	-
53	3-Ethylpentane	-	-	-	-	-	-	-	40.89±35.45
54	Aromatics	-	-	-	4.90±1.37	-	-	-	-
	**Other compounds**								
1	Benzoyl methamphetamine	-	-	36.96±18.08	11.01±1.75	-	-	-	-
2	Di-n-butyl ether	-	-	72.00±22.83	-	-	-	-	-
3	Furan	-	-	105.21±28.63	-	-	-	-	-
4	Acetonitrile-d3	-	-	45.60±27.02	-	-	35.5±20.8	-	-
5	Hydroxyacetonitrile	-	-	-	26.89±13.30	-	-	-	-
6	Heterocyclic compounds containing nitrogen	-	-	-	2.41±1.58	-	-	-	-
7	N-methyl-n-nitroso-ethylamine	-	-	-	-	1.63±1.22	-	-	-
8	Vinyl ether	-	-	-	-	8.83±3.10	-	-	-
9	Valeronitrile	-	-	-	-	3.86±1.38	-	-	-
10	2-cyanoguanidine	9.34±2.02	-	-	-	9.34±8.16	-	-	-
11	2-Hydroxypyridine	41.85±6.24	-	-	-	41.85±36.33	-	-	-
12	urrazole	-	-	-	-	2.68±1.32	-	-	-

13	2-methylaminomethyl-1, 3-dioxopentycline	-	-	-	-	6.90±3.01	-	-	-
14	2,3-dimethylpyrazine	-	-	-	-	4.95±1.64	2.62±1.28	-	5.49±3.91
15	n-Butyric anhydride	-	-	-	-	-	2.69±1.63	-	-
16	Acetic anhydride	-	-	-	-	-	28.7±11.0	-	-
17	1,2, 4-triazole [4,3-b] pyridine	-	-	-	-	-	22.9±10.4	-	-
18	4,5-dimethyl-1,3-dioxolan	-	-	-	-	-	0.71±0.26	-	-
19	2-methylpyrazine	-	-	-	-	-	-	-	5.14±4.51
20	tetramethylpyrazine	-	-	-	-	-	-	-	4.60±3.98
21	1,3-Di-tert-butylbenzene	15.94±9.52	18.52±6.63	12.10±5.70	50.22±4.39	-	-	-	-
22	p-Cresol	0.22±0.13	0.73±0.32	-	0.28±0.09	-	1.29±0.81	1.41±0.78	0.53±0.27
23	2,6-Dimethylpyrazine	-	-	223.5±28.3	16.94±3.95	20.40±13.20	31.9±5.51	20.2±11.75	28.0±8.38
24	Methyl Ether	51.85±7.89	24.89±9.12	-	99.53±26.09	89.89±23.49	185.21±78.9	94.26±32.48	197.8±56.91
25	N,N-Dibutylformamide	9.14±2.00	-	-	6.82±3.98	3.12±3.06	7.03±0.76	5.17±1.49	6.91±1.86
26	Methoxyphenyl oxide	1.00±0.85	0.85±0.41	-	-	0.31±0.19	-	-	-
27	DL-propyl ether lactam	1.02±0.86	1.86±0.44	-	-	-	-	-	-
28	pyrrole	0.86±0.35	6.55±0.73	-	0.86±0.25	-	-	-	-
29	2,3,5-trimethylpyrazine	-	2.70±1.56	16.12±11.03	-	12.10±9.07	22.7±3.33	15.24±9.17	27.57±19.18
30	Glycerin	2.36±0.48	1.48±0.39	-	-	-	6.68±2.85	3.08±1.96	-
31	n-Butylamine	-	-	6.45±2.47	-	-	-	-	-
32	(S) -L-alanine acetamide	-	-	-	-	6.38±5.59	-	-	-
33	Isobutyric anhydride	-	-	-	-	11.47±1.28	15.30±6.83	-	-
34	Butenyl propyl ether	-	-	1.21±0.16	-	-	-	-	-

Note: MA1: modified atmosphere packaging at 4 ℃ for one year, MA2: modified atmosphere packaging at 4 ℃ for two years, MB1: modified atmosphere packaging at -4 ℃ for one year, MB2: modified atmosphere packaging at -4 ℃ for two years;VA1: vacuum packaging at 4 ℃ for one year, VA2: vacuum packaging at 4 ℃ for two year, VB1: vacuum packaging at -4 ℃ for one year, VB2: vacuum packaging at -4 ℃ for two years, "-" means not detected.

Supplemental Table S2. Volatile flavor substance content statistics of Mianning ham in different packaging methods during storage stage.

Category	VA1		VA2		VB1		VB2		MA1		MA2		MB1		MB2		Total absolute content
	species	Absolute content(μg/kg)	species	Absolute content(μg/kg)	species	Absolute content(μg/kg)	species	Absolute content(μg/kg)	species	Absolute content(μg/kg)	species	Absolute content(μg/kg)	species	Absolute content(μg/kg)	species	Absolute content(μg/kg)	
**aldehyde**	3	143.98	6	309.25	2	10.2	8	144.95	9	419.52	7	150.31	3	30.49	7	269.69	1478.39
**ketone**	16	113.83	7	137.19	11	174.17	16	192.19	11	109.04	8	56.29	12	237.38	17	106.19	1126.28
**acids**	9	191.1	11	150.44	13	95.3	12	119.28	12	194.2	9	214.78	10	195.51	9	198.02	1358.63
**esters**	12	138.7	14	92.64	10	62.05	16	107.56	12	37.31	8	38.27	9	242.72	10	44.06	763.31
**alcohols**	19	522.78	20	461.26	17	320.22	28	393.43	15	290.33	13	145.71	11	94.91	19	547.61	2776.25
**hydrocarbon**	15	394.91	16	281.99	7	213.7	12	311.63	13	201.64	10	108.03	13	327.85	21	561.16	2400.91
**other**	15	223.71	13	363.23	6	139.36	8	276.04	10	133.58	8	57.58	9	519.15	9	214.98	1927.63
**total**	89	1729.01	87	1796	66	1015	100	1545.08	82	1385.62	63	770.97	67	1648.01	92	1941.71	11831.4

Note: MA1: modified atmosphere packaging at 4 ℃ for one year, MA2: modified atmosphere packaging at 4 ℃ for two years, MB1: modified atmosphere packaging at -4 ℃ for one year, MB2: modified atmosphere packaging at -4 ℃ for two years; VA1: vacuum packaging at 4 ℃ for one year, VA2: vacuum packaging at 4 ℃ for two year, VB1: vacuum packaging at -4 ℃ for one year, VB2: vacuum packaging at -4 ℃ for two years, "-" means not detected.
